# Are the Preliminary Impairment Tests used by UK police fit for purpose?

**DOI:** 10.1177/0025802420966402

**Published:** 2020-10-22

**Authors:** Gavin Trotter, Rhian Skinner, Brian Rooney

**Affiliations:** Applied and Human Sciences, Kingston University, UK

UK police officers make decisions about whether a person’s ability to drive properly is impaired. Those decisions are accepted by courts as evidence of driving-ability impairment. Those decisions are based on police officers’ subjective assessments of a set of tests that were not designed or validated to detect driving-ability impairment and with no established baseline of performance.

This editorial discusses the origins of the Preliminary Impairment Tests used in the UK to establish if a motorist’s ability to drive is impaired through alcohol or drug use. The focus of this editorial is how these impairment tests have never been validated against a control subject group, nor have they been validated to detect driving-ability impairment. A fundamental issue in this area is that the word ‘impairment’ is used extensively but not consistently in the scientific research and the legislation. It may refer to driving-ability impairment. It may refer to drug influence, which is impairment of the ability to perform tests designed to look for a drug. It may refer to physiological signs associated with drug use or to a specific blood-alcohol or drug concentration.

In the UK, section 5a of the Road Traffic Act 1988 sets per se limits for several drugs. Section 4 of the Road Traffic Act is an ‘impairment’ offence. It is an offence ‘to drive while unfit to do so through drink or drugs’, and a person is taken to be unfit if their ‘ability to drive properly is for the time being impaired’. To be unfit to drive, a person’s ability to drive must be impaired, and there must be a causal link between the impairment and any drugs found in their system. Similar laws exist around the world, pairing per se limit offences and impaired driving offences.

To assist in gathering evidence of driving-ability impairment, section 6B of the Act gives police the power to administer preliminary impairment tests. These tests, a pupillary examination and four divided attention psychophysical tests – the modified Romberg balance test, the walk-and-turn test, the one-leg stand test and the finger-to-nose test – are derived from the more extensive Drug Evaluation and Classification (DEC) programme from the USA.

Trained health-care professionals are also permitted to assess drivers using these impairment tests. However, following a reappraisal in June 2019, the Faculty of Forensic and Legal Medicine withdrew its support for the tests, stating on their website, ‘The Field Impairment Tests (FIT) have never been scientifically or statistically calibrated using a control group of subject drivers who had not taken any drugs’.^1^

The UK’s Preliminary Impairment Tests are derived from the American DEC system, itself an extension of the Standardized Field Sobriety Test (SFST) system, neither of which are designed or validated to detect driving-ability impairment. The SFSTs were originally developed as a predictor of blood alcohol concentration (BAC). The authors of the SFSTs are unequivocal that the system does not detect driving-ability impairment. The SFSTs detect alcohol influence on a subject’s ability to perform those tests, such that the operator can then estimate the subject’s BAC.

In a SFST study from 1998, it was noted that:

Many individuals, including some judges, believe that the purpose of a field sobriety test is to measure driving impairment … the developers of NHTSA’s [National Highway Traffic Safety Administration] SFST … pursued the development of tests that would provide statistically valid and reliable indications of a driver’s BAC, rather than indications of driving impairment.^2^

The purpose of the SFSTs was made clear in a study by Burns in 1974, which stated ‘we are detecting physiological changes, not impairment, as tolerance can result in people not being impaired at higher concentrations’.^3^

The DEC was originally developed from the SFSTs. What it is detecting is less clear. The training manual for DEC says:

… participants will learn to conduct systematic and standardized evaluations of persons suspected of drug impairment to determine:

(1) Whether the subject actually is impaired; and if so,

(2) Whether the impairment is drug- or medically-related; and if drugs,

(3) The category or combination of categories of drugs that is the likely cause of the observed impairment.

What does that actually mean? What impairment is being detected? To what purpose will the results of these tests be put? The DEC has been extensively validated for its ability to detect drug use and to determine what class of drugs have been used.^4^ The DEC only detects drug influence, meaning impairment of the ability to perform the tests; it does not assess driving-ability impairment. This issue was identified at an early stage in the development of the DEC^5^:

The DREs [Drug Recognition Experts] indicated whether they felt the suspects were ‘impaired’ by drugs (and hence ‘unable to operate a motor vehicle safely’) … There is no way to determine objectively whether the suspects were actually too ‘impaired’ to drive safely. The fact that drugs were found in a suspect’s blood does not necessarily mean the suspect was too impaired to drive safely.

The DEC emphasises that it is systematic and standardised. Officers are trained to look for specific clues in each test. In the walk-and-turn test for example, two or more clues, such as missing heel to toe or stepping off line, are an indicator of potential ‘impairment’. But again, impairment of what? In the SFSTs, two or more clues in the walk-and-turn test indicates a BAC ≥80 mg/100 mL (0.08%) with a 79% accuracy.^6^

In the UK, there are no assigned clue numbers for the tests, and while officers are trained to look for certain clues, ultimately they are left to make their own assessment as best they can. Police witness statements in drug-driving cases state:

I am an authorised Field Impairment Tester and I am aware of the codes of practice governing the administration of it. The purpose of it is to test whether a person is unfit to drive and if this is likely to be due to drink or drugs. It is not possible to ‘pass’ or ‘fail’ all or any one of the tests. There is no benchmark for pass or failure, nor is there any scoring system to indicate relative success.

Officers are simply left to make a subjective interpretation of what they see. An Australian study comparing officers’ assessments of potential driving-ability impairment using the SFSTs and the subjects’ performance in a driving simulator test found that officers’ decisions as to whether a person’s driving ability was impaired were wrong in between 25% and 30% of cases.^7^

The UK impairment tests require a set of validated studies that are linked specifically to driving-ability impairment. Future studies should establish a baseline performance of non-impaired drivers and more objective assessment criteria. In the absence of these changes, the UK Preliminary Impairment Tests remain a fundamentally flawed and deficient testing procedure.
